# Diet in Dyspepsia

**Published:** 1897-05

**Authors:** 


### DIET IN DYSPEPSIA.

Dr. A. L. Benedict in *American Therapist* says: As to dyspepsia, using the term somewhat vaguely, the writer can only reiterate what he has already written regarding dieting. It is the business of the physician not to reduce the diet to the level of gastric capability, but to elevate the gastric function to the requirements of normal food. The near-sighted patient pays the oculist for proper lenses, not for the advice to get near enough to objects to see them clearly; and the patient with dyspepsia is cheated, if the physician endeavors only to advise the use of foods which the stomach can digest in its weakened state. Diet in dyspepsia should be limited only temporarily or to a slight degree. It is proper enough to forbid such beverages as tea and coffee, which are positively, though mildly, toxic, and such achievements of culinary arts as cakes, pastry and heavy vegetables, which scarcely deserve the name of food. But almost all patients need some warm beverage, fruit, sugar, a variety of meats, breadstuffs, and concentrated vegetable nourishment.

Chocolate with milk, cocoa chips, or some of the cereal substitutes for coffee may be allowed as beverages.

As to fruits, a good general rule is to forbid the patient to swallow anything of the nature of skin, core or seed—with the possible exception of grapes, which are not palatable if the outer sweet coating is penetrated—but to allow a moderate quantity of the pulp or juice of any fruit desired, after meals.

There is one common fruit, namely, the banana,, which must be considered by itself. It is a large mass of raw starch, containing some sugar, and often an appreciable dose of nitrite of amyl. Physiologically speaking, it is rather a vegetable than a fruit; it is highly nutritious, but is not digested until the intestine is reached. Bearing these facts in mind, its indications and contra-indications can readily be determined; but the physician should remember, in all cases in which the subject of fruit is discussed with the patient, to except this one from the general category, and to explain that it is practically the same as a sweet potato.

Fruit should usually be freely allowed in conditions of constipation, and the existence of frequent cases of a typical scurvy must not be forgotten. The general malaise the dry and scaly skin, the aggravated indigestion of dyspeptics, who have been too rigidly dieted, are often, in the writer's opinion, scorbutic manifestations; and the same disease may certainly make itself apparent to a shrewd observer in artificially reared infants. Thus along with the infant's or the invalid's diet, when this is of a limited nature, there should be administered at least a little orange juice, or some similar antiscorbutic.

Sugar, or good candy, is essentially carbohydrate food in its most concentrated form. These foods do harm, usually because they are treated as offenders and are taken only after an abnormal appetite for them has been developed by too long abstinence. They should not be taken till the appetite for heartier food has been satisfied, and it must be remembered that craving for saccharine articles varies greatly with different individuals, and probably depends on individual differences in digestive chemistry, some digesting starches readily, others requiring sugars which conform more closely to the constitution of the digested molecule of carbohydrate food. The purest candy is that made at home by boiling down a solution of cane sugar, or by working confectioner's sugar into a paste and letting it harden. Various flavors, chocolate coatings, etc., may be added according to the taste of the patient. In patients with severe dyspepsia due to dilatation of the stomach, the writer has used a diet of pancreas sandwiches and sugar candy with much success.

In the selection of meats, we naturally turn first to beef, but idiosyncrasies must be respected. Some persons relish lamb, others poultry, a few even pork, better than beef. In conditions of true sub-acidity—the common cause or result of almost all cases of dyspepsia, whether organic or functional—there is at least a theoretical indication for sodium chloride and some antiseptic, which may be fulfilled by giving salted and smoked meats. Ham, crisp—not soggy—salt pork, raw salt codfish, perhaps slightly toasted, halibut, etc., may be proper food though not so quickly digested as fresh meats.

It is wise, whenever the patient is well enough to have his impressions and experiences considered, to ask him as to likes and dislikes, to be guided by his appetites and by his antipathies, to explain what foods he may take and why he may not take others, yet always to leave as wide a choice as possible and, even if the staple must remain the same, to vary the flavor from one meal to the next.

